# Reduced Cardiorespiratory Capacity in Children with Autism Spectrum Disorders

**DOI:** 10.3390/jcm7100361

**Published:** 2018-10-16

**Authors:** Véronique-Aurélie Bricout, Marion Pace, Léa Dumortier, Flavie Baillieul, Anne Favre-Juvin, Michel Guinot

**Affiliations:** 1 Université Grenoble Alpes, INSERM U1042, HP2, F-38000 Grenoble, France; ldumortier@chu-grenoble.fr; 2 UM Sports et Pathologies, CHU Sud, CS 90338, Avenue de Kimberley, F-38434 Echirolles CEDEX, France; afavrejuvinchu@gmail.com (A.F.-J.); MGuinot@chu-grenoble.fr (M.G.); 3 CHU de Grenoble, UF Recherche Exercice, UM Sports et Pathologies, F-38000 Grenoble, France; marion.pace01@gmail.com (M.P.); baillieul.flavie@gmail.com (F.B.)

**Keywords:** children, autism spectrum disorders, health assessment, physical fitness, motor impairments

## Abstract

**Background**—Children with autistic spectrum disorders (ASDs) are frequently hampered by motor impairment. It limits them from regularly practicing physical activities and results in a lower physical fitness even though low cardiorespiratory fitness is one of the most important predictors of all-cause mortality. This study aimed to investigate the cardiorespiratory fitness of boys with ASD compared to typically developed children. **Methods**—forty male children participated. Twenty were control children (CONT—10.0 ± 1.6 years) and 20 were ASD children (ASD—10.7 ± 1.2 years; intellectual quotient > 70). All participants completed an incremental exercise test on a treadmill. An evaluation of motor characteristics by three tests was conducted (muscular strength; explosive power; flexibility). Assessments of daily physical activity were obtained by questionnaires (PAQ-C) and by actigraphy. **Results**—in the ASD group, aerobic capacity values (VO_2peak_), effort duration and maximal speed were significantly lower compared to CONT (*p* < 0.05). Flexibility, explosive power and muscular strength were significantly lower in ASD compared to CONT (*p* < 0.05). Similarities between all children were observed for physical activity evaluation by actigraphy and with the PAQ-C. **Conclusions**—children with ASD had lower cardiorespiratory fitness than CONT despite similar physical activity levels. Our results suggested that the difference may be due to motor discrepancies.

## 1. Introduction

Autism spectrum disorders (ASDs) are referred to as a constellation of symptoms involving deficiencies in social interactions and communications, and restricted repetitive behaviors and interests. Many studies have reported the presence of motor and sensory difficulties in children with autism during their early development [1], and also the risk of inactivity in this population due to social and behavioral deficits [2]. These specific deficits could reduce possibilities for these children to participate in physical activity (PA), even though its importance to overall health for all subjects has been well documented [3]. Within this context, information about cardiorespiratory fitness evaluated by a standardized method in the laboratory in the ASD population is scarce, especially when we consider that impairments in motor skills are not diagnostic criteria for autism in children and adolescents.

Since many children with ASD lead a relatively inactive lifestyle, it has been proposed that an increase in physical activity might contribute to reduce the risk of mortality and morbidity associated with chronic disease in adulthood (cancers, cardiovascular disease, obesity [4]) or decrease functional difficulties such as stereotypical behaviors, hyperactivity, aggression and self-injury, and destructiveness [4].

The lack of research regarding physical activity patterns in children with ASD results in insufficient promotion and reduced involvement in sport for these children. Recognition of the benefits of physical exercise on psychological and physical health has given rise to specific guidelines for youth without disabilities such as daily 60-min appropriate moderate physical activity. These guidelines should also be applied to children with ASD, but it is not clear if these minimum standards are being met. In a study conducted at an elementary school by Pan [5], levels of physical activity obtained from actigraphy measurements were significantly lower for pupils with ASD than the control group. In order to encourage, adapt and secure the practice of sport, the physical fitness of the child to participate in sport activity should first be checked. According to the American College of Sports Medicine, measures of health-related physical fitness should include body composition, and cardiorespiratory and muscular fitness [6] where the cardiorespiratory assessment by a VO_2max_ measure is the gold-standard test [6].

The aim of this study was to investigate in the laboratory the physical fitness of children with ASD compared to typically developed children, measuring the maximal rate of oxygen uptake (VO_2max_).

## 2. Experimental Section

### 2.1. Study Population

The study was approved by the Ethics Committee of the Hospital of Grenoble (France) (N°A00-865 40) and was registered on the Clinical Trials Gov. Registry, with the number NCT 02830022 (12 July 2016). All experiments were performed in accordance with relevant guidelines and regulations. Each parent and child received information about the purpose and nature of the protocol. After reading and understanding the information, a statement attesting to informed consent was approved, obtained and signed by the child and by his parent and/or legal guardian.

Forty male children (10.4 ± 1.5 years, range 8 to 13) participated and were recruited from local support groups (institution specialized in care for children with ASD or public school, or association of parents). All were Caucasian. Twenty were typically developed children and served as controls (CONT) and 20 were children with ASD (ASD). All children attended regular classes in mainstream schools. All children with ASD normally participated in all school activities including school sports activities.

Diagnosis of ASD was certified by experienced physicians and psychologists, according to the DSM-V [7]. The children were also evaluated with the Autism Diagnostic Observation Schedule (ADOS) [8]. Intellectual quotient (IQ) was assessed using the Wechsler Intelligence Scale for Children, 4th Edition [9]. The inclusion IQ criterion was children with IQ > 70 (children with intellectual disabilities (IQ < 70) were not included). In respect with ethical guidelines, IQ scores and ADOS results had not been transmitted to researchers. Nevertheless, IQ scores were guaranteed as being >70 by a clinical psychologist experienced in diagnosing children with ASD. Diagnoses of autism were confirmed for all ASD subjects participating in this study. In addition, the Vineland scale-II was completed by parents [10]. This scale assesses three dimensions of adaptive daily behaviors (communication, daily living skills and socialization).

Other exclusion criteria of the participants were: (i) contra-indication against physical exercise (e.g., no cardiovascular disease or cardiac insufficiency that might alter heart rate (HR) response); (ii) psychiatric disorders or comorbid medical (e.g., no epilepsy); (iii) severe osteo-articular pathology; (iv) respiratory disorders (e.g., asthma); and (v) medical treatment that may alter the metabolic response. One child was excluded because he refused the application of the electrodes for the electrocardiogram (ECG) indispensable to monitor the maximal treadmill test. The characteristics of the two groups are presented in Table 1.

Body mass index was calculated (BMI: body weight in kg/height in m^2^) and pubertal stage of the children was assessed by Tanner criteria [11]. Each child underwent a medical examination and a 12-lead resting electrocardiogram (ECG) after 5 min in supine position (HR_rest_) prior to the treadmill test to assess the absence of contra-indication.

### 2.2. Incremental Exercise Test

Each child had realized a continuous maximal incremental protocol, walking on a treadmill (Gymrol Super 2500, Andrézieux, France). After adjustment to an easy walk (starting at 3 km·h^−1^ for children 8 to 10 years old, and 4 km·h^−1^ for children 10 to 12 years old), the speed and the slope of the treadmill were alternatively increased by 0.8 km·h^−1^ or 2% every minute until exhaustion. During this maximal test, gas exchanges were measured continuously on an ergospirometer (Brainware Metasys, France). Both pneumotachograph and gas analyzers were calibrated before each test.

Heart rate was continuously monitored and displayed on a 12-lead ECG (PC-ECG 1200 Norav Medical, Germany) and HR_peak_ was obtained during this maximal test. Predicted peak HR was defined as (220—age) [12].

Children who completed at least 3 out of the 4 following criteria during protocol test were considered to have achieved a maximal VO_2_ test and reached their VO_2peak_: *(a)* maximal heart rate >90% of predicted HR_peak_; *(b)* respiratory quotient of ≥1.01; *(c)* achievement of an oxygen plateau (defined as a ≤2.0 mL/kg per min change in oxygen uptake during the last minute of test); *(d)* incapacity to follow the treadmill speed despite verbal encouragements [6,13]. In addition to these cardiorespiratory markers, maximal characteristics of exercise were assessed by blood lactate measure with 20 μL of arterialized blood sampled at a fingertip during the 4th min of recovery.

The perception of fatigue was assessed with a visual scale in order to verify the apparition of fatigue during the effort.

### 2.3. Physical Activity Assessment

Physical activity was evaluated using the SenseWear^®^ accelerometer Pro-3 (SenseWear Armband Bodymedia, Pittsburgh, PA, USA) over a seven-day period during a typical school week. Participants wore the monitor on the triceps (midhumerus point) using an elastic belt. The monitor had to be worn for all activities except showering or swimming, and it must be held in place 24 h a day. All participants were asked to wear the monitor for every activity of the day (school, leisure, physical education etc.), every day of the week as well as during the weekend, and for PA during and after school. A daily diary was completed by parents in order to record precise moments when the child did not carry the accelerometer (i.e., shower, nautical activity etc.). Such non-wear time was removed from the analysis. Raw accelerometer counts were downloaded using Sense Wear software (version 7.0) and served to calculate the time spent in sedentary behavior or in PA. Parents and children completed the Physical Activity Questionnaire for Children (PAQ-C) [14]. The PAQ-C includes 9 items to calculate a total PA score. It determines the periods of PA during the week or the weekend, and with or without a teacher.

### 2.4. Motor Tests

In consistency with the recommendations of the American College of Sports Medicine, health-related physical fitness includes body composition, aerobic capacity, flexibility and muscular strength [6]. With reference to the previous work with this population [15], physical fitness was evaluated with three tests from the EUROFIT Physical Fitness Test Battery. All fitness evaluations were carried out by an experienced exercise physiologist. Children performed all tests in standardized conditions. Verbal explanations and/or pictures and/or demonstrations were used—if necessary—to help the children to understand the tasks. The child was tested individually by the same experienced therapist. Maximal treadmill test was always done first, followed by a rest of 15–20 min, and then the motor assessment session. There was no precise order for completion of the motor tests, they were independent from each other. Specifically, muscular strength was evaluated by the handgrip test measuring the maximum isometric strength of the hand and forearm muscles [16]. The child held the dynamometer (SMFD500TR1300N, Sensel, France) in the preferred hand to be tested. When ready, he squeezed with maximum isometric effort the dynamometer. Handgrip measures the maximal amount of tension that could be applied in a single muscular contraction. The value was registered by a score on a digital sensor (PAXS0000, Sensel, France) in arbitrary unit (a.u). Another test (standing broad jump) measured the explosive power of the legs. The child made a jump as far as possible. The maximum horizontal distance reached was measured in centimeters [16]. The sit and reach flexibility test is a measure of flexibility of the lower back and hamstring muscles [16]. The child—sitting on the floor with knees extended—was asked to bend the trunk and reach forward as far as possible. The line of the toes represented the zero point. Any measure beyond this line was positive and below the measure was negative. The score was expressed in cm and higher scores indicated better flexibility.

### 2.5. Statistical Analysis

Data are expressed as means +/− standard deviations. A Kolmogorov–Smirnov test allowed us to verify the normality of the distribution. Our data were not normally distributed, so to compare ASD and control groups we performed a Mann–Whitney U test (Statistica Software 8.0, TIBCO, Tulsa, OK, USA). Relations between variables were obtained by Spearman rank order correlations (Statistica Software 8.0). Significance was accepted when *p* < 0.05 after adjustment by using the Bonferroni correction.

## 3. Results

Participants’ characteristics are shown in Table 1. There was no statistical difference in anthropometric characteristics between the two groups (Table 1). Vineland scale results showed that children with ASD had lower scores than control children in all dimensions (Table 1), confirming the significant alterations of adaptive behavior in the ASD group.

The results of the maximal treadmill test are displayed in Table 2.

The ASD group had a lower VO_2peak_ than the controls (*p* < 0.05—Table 2), and showed lower effort duration, maximal speed and treadmill slope compared to control (*p* < 0.001—Table 2). No difference existed between ASD and control on other cardiorespiratory and metabolic data. One child with ASD and one control only reached two out of four standard criteria of a maximal VO_2_ test.

Flexibility, explosive power and muscular strength were significantly lower in ASD compared to controls (*p* < 0.05—Table 2). No correlation was found between VO_2_ and flexibility or strength measures (respectively r = 0.12; r = 0.17 and r = 0.10; NS).

Concerning the PA evaluation by actigraphy and PAQ-C (Table 3), no significant difference between the two groups was observed. No correlation was found between cardiorespiratory or motor variables and PA data. In the ASD group, sedentary time score (day/min) was significantly correlated with global score of PAQ-C (r = −0.78, *p* < 0.001), therefore, the lower the PAQ-C score, the higher the sedentary time.

## 4. Discussion

The aim of this study was to investigate the physical fitness of children with ASD compared to typically developed children. The current study showed that children with ASD have specifically lower VO_2peak_ compared to controls, with significantly impaired running performance during the maximal treadmill test (final slope, speed and effort duration). To our knowledge, this is the first report on cardiorespiratory fitness evaluated by a gold-standard measurement and a direct assessment of the maximal oxygen consumption during exercise testing (VO_2_) in children with ASD. Literature that compares the level of physical fitness and activity of children with ASD to children without ASD is scarce, even though there are studies that have reported results with specific tests such as those used in the evaluation by EUROFIT [15,18,19] and activities evaluated by questionnaires or actigraphy [20,21,22]. These studies have reported high difficulties in balance, postural stability, movement speed and strength in the ASD group [15,18,19]. These deficits in motor abilities may be exacerbated because of reduced opportunities to engage sufficiently in physical activity, leading to the adoption of a sedentary lifestyle, and consequently a low level of physical fitness. Therefore, developing motor capacities is essential because this contributes to improve cardiorespiratory health. In this context, Tyler et al. [23] have reported some results on the physical activity and fitness of children with ASD, and they have shown that children with ASD are less physically active and fit than typically developed children. In this study, authors have reported an estimated maximal aerobic power assessed using the 20-m multistage shuttle run, and they did not observe a significant difference on estimated VO_2_ values between controls and children with ASD. However, in this study, the VO_2_ value was not measured by a direct method, but was estimated from a predictive equation, that could induce skewed results. A recent review written by Srinivasan et al. [24] reported results of studies focusing on physical fitness in ASD. From these studies, six proposed a VO_2_ assessment obtained by indirect techniques or by predictive equation. Although this VO_2_ method provides a correct evaluation of the cardiorespiratory fitness range of the participant, it has not the same accuracy as values obtained with a gas-exchange measurement. Overall, conclusions drawn from empirical evidence on the efficacy of physical activity interventions to improve cardiorespiratory fitness in individuals with ASD, although promising, are currently limited due to the lack of rigorous methods.

Because movements are difficult and physical activity less important for children with ASD, they could be less likely to be physically active. Accordingly, they have less developed physical fitness and motor skills [25]. In our study, cardiorespiratory fitness is decreased (but nevertheless at a good level) in children with ASD compared to controls even though our two groups are matched in age, height, weight and gender. There was no significant difference in the amount of physical activity between children with ASD and controls in our study that could account for the VO_2_ difference between the two groups (Table 3) at the time of the study.

The effort duration, maximal speed and slope of treadmill reached at the end of the test were significantly lower in the ASD group. This was not the consequence of a cardiorespiratory maladaptation, as HR_peak_, ventilation and respiratory exchange ratio were not significantly different between the two groups. Moreover, it is not a metabolic adaptation problem, as lactatemia was not significantly different between CONT and ASD children. This could be a consequence of a limited capacity of the children with ASD to sustain the physical effort with increasing exercise difficulty.

The VO_2max_ value achieved during a progressive maximal protocol to voluntary exhaustion has been considered for a long time by the World Health Organization as the best indicator of cardiorespiratory fitness [26]. In this context, the maximal oxygen consumption can be evaluated by maximal or submaximal test, using direct or indirect methods. The most widely used examinations are walking/running tests in the laboratory or the 20-m shuttle run test (as proposed by Tyler et al. [23]). We chose a treadmill ergometer because it is generally considered superior (approximately 8% better) to bicycle ergometers for the assessment of VO_2max_ in children [27]. Moreover, this choice is also supported by the fact that in young children, treadmill testing as well as walking on a treadmill is a more natural activity than pedaling while maintaining a cadence, and is less affected by factors such as lack of skill, motivation and local muscle fatigue [27].

Furthermore, participants from our two groups (except for one in each) completed a continuous incremental treadmill protocol until termination criteria had been reached, and with voluntary cessation despite the encouragements. According to the standard criteria, we can assume that these factors could not explain the VO_2max_ difference between ASD and control children. Moreover, no significant difference was found in blood lactate concentration values between the two groups after the test, and this confirms that metabolic factors cannot explain the VO_2max_ difference. We did not retain the lactatemia as a standard criterion of a maximal effort in adults, as pediatric literature is not unanimous on the maximal blood lactate concentration. The lower values of lactatemia in children after a maximal exercise reflect a lower extravascular rise in lactate produced by the muscular metabolism during exercise combined with faster turnover of the blood lactate from the intravascular compartment in children compared with adults [28], and this parameter cannot therefore be retained as a variable of maximal glycolytic metabolism in children. Finally, other factors could explain these lower VO_2peak_ values in ASD children.

The lower VO_2peak_ values in the ASD group could be related to motor disorders classically described in autism [1,29,30,31,32]. These authors reported the presence of sensory-motor deficits (e.g., gross and fine motor problems), impairment in movement/motor skills, balance deficits, muscle weakness and hypotonia when compared to children without ASD. Moreover, several authors have reported the presence of dyspraxia in children with ASD that could increase these motor disorders [33,34]. Some motor difficulties are significant in our ASD group who had lower performances on flexibility (*p* < 0.05), explosive strength (*p* < 0.05) and muscular strength (*p* < 0.05) than the control group. These observations are concordant with others [15,18,23] and could explain the lowest values of speed, slope and duration of effort in ASD. Children with ASD with less strength would also be more quickly limited to pursue an effort, and would be more quickly tired than controls.

Other studies have reported some disturbance of regular, rhythmic and automatic locomotor pattern maintenance in the ASD population (shortened stride length, elongation of cadence, of wide base of support) and increased variability that indicate a high-level difficulty in integrating information to regulate movement. In this work, we analyzed with an Optogait device (Microgate, Italia; data not shown) the first minute of walking on the treadmill, and no difference between our groups was found. Therefore, this factor cannot explain the VO_2_ difference in this experimental context. However, this result is to be nuanced because on the treadmill, the walking pattern is no longer natural, and during the first minute there is an adaptation time that is possibly the same for all children with or without ASD. We also calculated the energy cost during this treadmill test, and the lack of significant difference between the two groups could not explain the VO_2_ difference.

It therefore seems essential to identify another reason to explain this difference. Motivational behavior could be one of the causes. If the child does not like to participate in physical activities, he will be less motivated to perform the exercise, and a child with ASD may be less likely to respond to social reinforcement or encouragement. In order to avoid this phenomenon, we used pictograms during the test to allow the child to visualize the objective to reach. Furthermore, we continuously encouraged every child during the test.

It is also possible that the volume of physical activity practiced since early childhood is a probable explanatory factor. Indeed, VO_2max_ is a parameter that evolves with regular and sustainable training. Thus, if a child is motivated by a sporting practice and develops a high level of competence in this activity, then the VO_2max_ value is likely to be higher. The child’s sports environment is an essential determinant of physical fitness. Children with autism have greater difficulties than typically developed children with participating in physical activity because of the nature of their motor and environmental difficulties. Some studies have found that children with autism are less active than children without disabilities [35]. It is possible to reduce the PA practice because of an existing motor impairment that causes less opportunities to develop and enrich existing and new physical abilities. This observation could corroborate this VO_2_ difference. In our study, the results of physical activity quantification (Table 3) showed that in every category, control children had higher activity (even though not significant), and confirmed the results obtained by Dickinson et al. [35]. These authors indicate that an improvement in PA can be achieved by children with ASD if they follow a computer-based activity program, and so using a booster. Conversely, Tyler et al. [23] have shown that children with ASD are less physically active than typically developing peers, a result similar to those published previously [20,21] but different from our study. However, in these studies [20,21,23], children were diagnosed with a moderate-to-severe ASD, and recruited for an interventional study to propose a training program. Therefore, these participants were likely to have a lower level of physical activity and this represents a recruitment bias compared to our study. Moreover, Mac Donald et al. [21] have shown that daily PA became less prominent when age increased. Indeed, teenagers (14–18 years old) were significantly less active than younger children with ASD (9–11 years old). In our study, children were younger (mean age: 10 years old) and all active, which may partly explain these differences. These trends might be significant with larger samples, and might explain some of the VO_2_ difference.

Moreover, our results indicated high VO_2peak_ values in the two groups, but we have confidence in the accuracy of these values because we used rigorous methods to measure cardiorespiratory fitness during the treadmill test, and these values are close to those predicted in young sedentary males on a treadmill test [36].

Finally, some limitations still need to be taken into account. Firstly, in order to increase statistical power and confidence in the generalizability of the results, related to PA measures, there will be a need of a higher recruitment of children. Secondly, it will be necessary to reproduce the same study with children with more severe ASD because in this work, only boys with IQ > 70 were included. Thirdly, future research should also consider the use of samples with girls to allow for further subgrouping, examining older or younger children with ASD, and considering the use of diverse physical activity measures as outcome measures for interventions designed to address the specificity of children with ASD.

## 5. Conclusions

This study reports new results showing a lower physical fitness in children with autism. Our results may indicate significant underlying deficits in cardiorespiratory and muscular fitness, essential parameters for the production of controlled and good-quality movement. These differences were not explained by a lower physical activity level or a lack of motivation to take part in the protocol. These results suggested that children with ASD are at greater risk for poor aerobic and muscular fitness in adulthood than their unaffected peers, especially as this study involved fit and physically active ASD children. As lower physical fitness is a reliable indicator for poor health outcomes, this study provides an important argument for the systematic implementation of physical activity programs for children with ASD.

## Figures and Tables

**Table 1:** Participants’ anthropometric characteristics and Vineland assessment.

	CONT	ASD
Age (years)	10.0 ± 1.6	10.7 ± 1.2
Weight (kg)	33.3 ± 7.2	33.5 ± 5.3
Height (cm)	141.0 ± 10.5	144.2 ± 7.4
BMI (kg m^−2^)	16.0 ± 1.5	16.6 ± 1.6
Ratio waist/hip	0.88 ± 0.05	0.86 ± 0.05
Tanner stage 1 (no. of subjects)	16	15
Tanner stage 2 (no. of subjects)	4	5
Vineland assessment		
Communication	121 ± 5	106 ± 11 ***
Daily living skills	132 ± 12	116 ± 14 ***
Socialization	106 ± 9	88 ± 13 ***

Values are means ± SD. BMI: body mass index (see method section). Tanner stage: number of subjects in this stage. a.u: arbitrary unit. Low negative values of flexibility represent a low performance. Strength: the higher the value, the higher the performance. Significantly different from CONT * *p* < 0.05; ** *p* < 0.01; *** *p* < 0.001.

**Table 2:** Results of maximal treadmill test, motor assessment data.

	CONT	ASD
HR_rest_ (bpm)	76 ± 10	76 ± 8
HR_peak_ (bpm)	194 ± 15	193 ± 14
Predicted peak HR (bpm)	209 ± 1	209 ± 1
%HR_max_ > 90% predicted HR	92.1 ± 7.1	91.7 ± 4.5
HR_post+5min_ (bpm)	106 ± 13	106 ± 10
VO_2peak_ (mLO_2_·kg^−1^·min^−1^)	58.1 ± 8.8	52.1 ± 6.4 **
Predicted peak VO_2peak_ (mLO_2_·kg^−1^·min^−1^)	52.5 ± 1.1	51.5 ± 0.9
%predicted VO_2peak_	111 ± 17	100 ± 12 *
VE_peak_ (L·min^−1^)	64.2 ± 15.0	62.9 ± 10.7
RER	1.12 ± 0.08	1.16 ± 0.08
Maximal treadmill slope (%)	15.7 ± 2.8	12.1 ± 2.6 ***
Maximal speed (km·h^−1^)	17.2 ± 2.1	14.8 ± 1.8 ***
Effort duration (min)	13.0 ± 1.7	11.0 ± 1.4 ***
Energy cost (mLO_2_·kg^−1^·m^−1^)	0.20 ± 0.03	0.21 ± 0.02
Lactatemia (mmol·L^−1^)	6.13 ± 2.65	5.01 ± 1.98
Perception of fatigue	2.90 ± 0.64	2.90 ± 0.97
Flexibility (cm)	−16.4 ± (−8.3)	−22.2 ± (−7.1) *
Explosive power (cm)	132.0 ± 22.6	117.3 ± 21.5 *
Muscular strength (a.u.)	162 ± 51	129 ± 39 *

Values are means ± SD. HR: heart rate in beats per minute; VO_2_: oxygen uptake; VE_peak_: peak ventilation; energy cost = (VO_2max_ − 0.083) × Speed_max_^−1^ (m·s^−1^) [17]; RER: respiratory exchange ratio; perception of fatigue, see methods. Significantly different from CONT * *p* < 0.05; ** *p* < 0.01; *** *p* < 0.001.

**Table 3:** Results of physical activity evaluation by actigraphy and questionnaire.

	CONT	ASD
Total physical activity time (min/day)	202 ± 60	181 ± 84
Sedentary time (min/day)	600 ± 82	624 ± 104
Time in moderate activity (min/day)	155 ± 55	146 ± 67
Time in vigorous activity (min/day)	35 ± 17	24 ± 24
Time in very vigorous activity (min/day)	7 ± 7	3 ± 5
Total recording time (h/24 h)	23 h 21 min	23 h 29 min
PAQ-C; global score	3.1 ± 0.6	2.7 ± 0.8

Values are means ± SD. PAQ-C: physical activity questionnaire for children. The average time spent in sedentary behavior and during various PA is presented in min/day. No significant difference between groups.
